# Parental knowledge and acceptance of routine childhood immunization: Association with verified vaccination status - A cross-sectional study

**DOI:** 10.6026/973206300222025

**Published:** 2026-04-30

**Authors:** Jeevitha Shetty Solur Venkatesh, Leena Balpande

**Affiliations:** 1 Department of Medicine, Sapthagiri Institute of Medical Science, Karnataka, India; 2 Department of Community Medicine, NKP Salve Institute of Medical sciences & RC and LMH, Nagpur, India

**Keywords:** Parental awareness, vaccine acceptance, childhood vaccines, vaccination status of children, routine vaccines, immunization coverage, vaccine hesitancy, public health, health education, parental attitude

## Abstract

Incomplete childhood immunization remains a preventable contributor to morbidity despite established national vaccination programs. Therefore, it is of interest to evaluate parental knowledge and acceptance of routine childhood immunization and examined their association with verified vaccination status among 423 caregivers of children aged 0-59 months. Although 86.3% were aware of the national immunization program and 91.7% believed vaccines are necessary, only 46.8% correctly identified all vaccine-preventable diseases. Verified records showed 78.0% full immunization and higher parental awareness was strongly associated with complete vaccination (AOR 3.25, p < 0.001). Thus, we show that knowledge gaps and misconceptions persist which significantly influence timely immunization completion.

## Background:

Childhood immunization is a cornerstone of public health and prevents substantial morbidity and mortality worldwide [1]. Despite the availability of effective vaccines, gaps in routine immunization coverage persist in many regions [2]. Incomplete vaccination exposes children to preventable infectious diseases and undermines herd immunity [3]. Parental decision-making plays a central role in childhood vaccine uptake. Knowledge of vaccine schedules, perception of safety and trust in healthcare systems influence adherence to immunization programs [4]. Vaccine hesitancy, driven by misinformation and fear of adverse effects, has emerged as a growing public health concern. Misconceptions regarding immune overload and vaccine safety contribute to delayed or incomplete vaccination [5]. Socio-demographic determinants such as maternal education, household income and access to healthcare services also affect immunization status [6]. Health worker counselling remains a strong predictor of vaccine acceptance. However, awareness does not always translate into completed immunization schedules. Self-reported vaccination status may overestimate true coverage due to recall bias [7]. Verification of immunization records provides a more objective measure of vaccine uptake. Correlating parental knowledge and acceptance with documented vaccination status enables assessment of whether awareness results in preventive action. Identifying modifiable determinants of incomplete immunization is essential for strengthening national immunization programs [8]. Therefore, it is of interest to evaluate parental knowledge and acceptance of routine childhood immunization and examine their association with verified vaccination status.

## Materials and Methods:

A cross-sectional questionnaire-based study was conducted in the Department of Pediatrics of a tertiary care centre in India between January and June 2024. Parents or primary caregivers of children aged 0-59 months attending the pediatric outpatient department or immunization clinic were eligible. The sample size was calculated assuming 50% prevalence of adequate parental knowledge, with 95% confidence level and 5% margin of error, yielding a minimum of 384 participants. After accounting for 10% non-response, a target sample of 423 was determined. Participants were recruited using consecutive sampling until the required sample size was achieved. A pre-tested interviewer-administered structured questionnaire in the local language assessed socio-demographic characteristics, knowledge of the immunization schedule, attitudes toward vaccines, reasons for hesitancy and sources of information. The tool was pilot tested among 30 caregivers and demonstrated acceptable internal consistency (Cronbach's alpha = 0.82). Vaccination status was verified using immunization cards when available and by caregiver recalls when documentation was absent. Data were entered into a secured database and analysed using descriptive statistics. Associations between categorical variables were examined using chi-square tests. Binary logistic regression analysis was performed to identify independent predictors of complete immunization. Adjusted odds ratios with 95% confidence intervals were reported and p < 0.05 was considered statistically significant. Ethical approval was obtained from the Institutional Ethics Committee and written informed consent was secured from all participants.

## Results:

A total of 423 parents or caregivers participated in the study. Most respondents were aged 25-34 years and females constituted the majority. Educational attainment was high, with over 80% completing secondary education or higher. Awareness of the national immunization program and the importance of completing all vaccine doses were high. However, knowledge of all vaccine-preventable diseases was substantially lower. Parental attitudes toward vaccination were generally positive, although misconceptions regarding immune overload persisted. Fear of side effects was the leading cause of vaccine hesitancy. Health workers were the primary source of vaccine-related information. Verified immunization records showed higher full immunization rates compared to parental recall. Complete immunization was significantly associated with higher awareness levels and maternal education. Logistic regression identified high awareness, maternal education and access to health workers as independent predictors of full immunization. Overall KAP scores indicated moderate-to-good knowledge and positive attitudes. Table 1 (see PDF) shows that 47.5% of respondents were aged 25-34 years, 59.3% were female and 80.6% had secondary education or higher. Table 2 (see PDF) indicates that awareness was highest for the national immunization program at 86.3% and lowest for knowledge of all vaccine-preventable diseases at 46.8%. Table 3 (see PDF) demonstrates that 91.7% agreed vaccines are necessary and 83.2% considered them safe, while 27.9% believed multiple vaccines could weaken immunity. Table 4 (see PDF) highlights that fear of side effects accounted for 39.3% of hesitancy cases, followed by lack of schedule awareness at 24.3%. Table 5 (see PDF) compares information sources and shows that 44.9% relied on health workers, followed by 23.2% consulting doctors or nurses. Table 6 (see PDF) depicts full immunization in 83.1% of card-verified cases compared with 64.3% based on recall, with overall full immunization at 78.0%. Table 7 (see PDF) shows a statistically significant association between high awareness and complete immunization, with 90.1% fully immunized in the high-awareness group. Table 8 (see PDF) indicates that full immunization increased from 58.5% among primary-educated parents to 89.7% among graduates. Table 9 (see PDF) demonstrates that high parental awareness (AOR 3.25), maternal education (AOR 2.48) and access to health workers (AOR 2.15) were independent predictors of complete immunization. Table 10 (see PDF) highlights an overall mean KAP score of 23.6 ± 4.8, indicating moderate-to- good parental knowledge and positive attitudes.

## Discussion:

This study evaluated parental knowledge and acceptance of routine childhood immunization and examined their association with verified vaccination status. The findings demonstrate generally positive attitudes toward vaccines but persistent knowledge gaps and measurable discrepancies in immunization completion [9]. Although awareness of the national immunization program was high, detailed understanding of vaccine-preventable diseases and booster requirements remained limited [10]. The observed full immunization rate of 78% indicates moderate coverage. This level remains below optimal thresholds required for sustained herd immunity. Verified card-based immunization was substantially higher than recall-based reporting. This discrepancy highlights the limitation of relying solely on parental recall in coverage assessments. Documentation verification strengthens accuracy and reduces overestimation bias [11]. Knowledge deficits were evident despite high acceptance. Nearly one-third of parents believed that multiple vaccines could weaken immunity. Such misconceptions reflect persistent misinformation. Fear of side effects was the leading cause of hesitancy. These findings align with contemporary research indicating that safety concerns remain a dominant barrier to vaccine uptake [12]. Parental awareness demonstrated a strong independent association with complete immunization. Caregivers with high awareness were more than three times as likely to have fully immunized children. This finding underscores the behavioural influence of knowledge on preventive health decisions. Awareness facilitates timely compliance with immunization schedules when reinforced through credible information sources [13]. Maternal education emerged as a significant predictor of full immunization. Children of mothers with secondary or higher education showed substantially higher coverage rates. Education enhances health literacy, risk perception and engagement with healthcare services. These findings are consistent with global evidence linking maternal education with improved child health outcomes [14]. Access to health workers significantly influenced immunization status. Health workers were the primary source of vaccine-related information. Their role extends beyond service delivery to counselling and trust-building. Structured communication by frontline health personnel strengthens parental confidence and counters misinformation. This emphasizes the importance of sustained provider engagement in routine pediatric visits [15]. Despite high agreement that vaccination is necessary, awareness gaps suggest incomplete understanding of schedule complexity. Booster dose awareness was moderate, indicating the need for reinforced schedule education. Partial immunization may reflect missed opportunities during follow-up visits rather than outright refusal [16]. The study contributes methodological advancement by correlating parental KAP assessment with verified immunization records. Few cross- sectional studies integrate behavioural evaluation with objective verification. This dual approach enhances internal validity and clarifies whether awareness translates into documented preventive action. The findings demonstrate that positive attitudes alone are insufficient without adequate knowledge depth [17]. Limitations should be acknowledged. The cross-sectional design precludes causal inference. Participants were recruited from a tertiary care centre, which may overrepresented health-conscious caregivers. Recall bias may persist in participants without documentation. However, the inclusion of immunization card verification mitigates reporting inaccuracies. Public health implications are clear. Educational strategies must move beyond general promotion of vaccination to targeted clarification of vaccine-preventable diseases and booster schedules. Behaviour-change communication should address specific misconceptions regarding immune overload and safety concerns. Provider-led counselling remains central to improving compliance. Overall, parental awareness and maternal education significantly influence complete childhood immunization. Verified record analysis confirms that knowledge correlates with preventive behaviour. Strengthening structured health education and reinforcing provider communication are critical to improving routine immunization coverage.

## Conclusion:

Parental knowledge and acceptance significantly influence completion of routine childhood immunization. Verified vaccination status demonstrates that higher awareness and maternal education are independently associated with full immunization. Persistent misconceptions and knowledge gaps continue to affect schedule compliance. Thus, strengthening structured counselling and improving awareness of vaccine- preventable diseases are essential to sustain optimal immunization coverage.
